# Lions select larger prey in a Central African protected area with increasingly effective management

**DOI:** 10.1002/ece3.70062

**Published:** 2024-07-21

**Authors:** Chiara Fraticelli, Abdoulaye Abakar Zayed, Herwig Leirs, Hans Bauer

**Affiliations:** ^1^ Evolutionary Ecology Group, Department of Biology University of Antwerp Antwerp Belgium; ^2^ African Parks Network, Greater Zakouma Ecosystem Zakouma Chad; ^3^ Wildlife Conservation Research Unit, Biology University of Oxford, the Recanati‐Kaplan Centre Tubney House Abingdon UK

**Keywords:** carrying capacity, delegated management, lion, *Panthera leo*, prey preference, Zakouma National Park

## Abstract

Lions and their prey are threatened across most of their range and especially in West and Central Africa. Prey availability influences carnivore densities, social structure, prey preference and home ranges, and changes in prey are important for carnivore management. Scarcity of large prey in many West and Central African ecosystems has been described as leading to a preference for hunting smaller prey in smaller groups. Here we investigated the changes in prey selection of lions in Zakouma National Park (Chad), a protected area in Central Africa that showed significant recovery in wildlife numbers, by collecting feeding data through observations of lions on kills during monitoring drives and GPS cluster points of lion collars. Compared to similar data collected prior to this significant recovery, lions preferred larger prey and fed in larger groups. Our results show that diet shifts due to prey losses can be reversed with restoration of prey populations thanks to improved management, and we speculate that this may be true across large carnivores and across regions.

## Graphical abstract

Lions and their prey are threatened across most of their range and especially in West and Central Africa, where scarcity of large prey has been described as leading to a preference for hunting smaller prey in smaller groups. We investigated the changes in prey selection of lions in Zakouma National Park (Chad) and our results show that diet shifts due to prey losses can be reversed with restoration of prey populations.

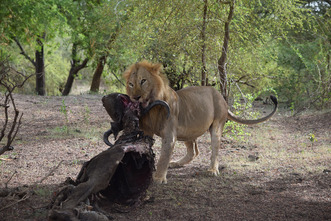

## 1 INTRODUCTION

Wildlife in the Sudan‐Sahel belt across West and Central Africa is threatened; between 1970 and 2005, large mammals in protected areas in West Africa declined by 85% (Craigie et al., 2010). In Central Africa wildlife declined less dramatically until the turn of the century, but accelerated in the last 20 years (Scholte, 2011). Lions (*Panthera leo*) are declining in most regions of Africa, except for intensively managed areas (Bauer et al., 2015; Lindsey et al., 2017; Packer et al., 2013). In West Central Africa, lions have gone extinct or near‐extinct in 61% of the protected areas where they occurred historically (Brugière et al., 2015), and in most of the areas where they are still present they are at less than 50% of carrying capacity (Lindsey et al., 2017), and declining in all studied protected areas apart from Pendjari National Park (Benin) (Bauer et al., 2015).

Our current understanding of lion feeding ecology in the region is mostly based on a few studies in degraded landscapes. In West and Central Africa large prey form a significantly lower percentage of lion kills compared to South and East Africa, but this is attributed more to availability of species than preference (Bauer et al., 2008). In Comoé NP (Cote d'Ivoire) and in Faro NP and surrounding hunting areas (Cameroon), Buffon's kob (*Kobus kob*) was the species most frequently killed by lion, followed by bushbuck (*Tragelaphus scriptus*) (Bodendorfer et al., 2006; Breuer, 2005). Large prey (>250 kg) only constituted 3.8% of kills in Comoé NP and 7.5% in Faro NP. In Waza NP (Cameroon) kob was again the most frequent prey species, however due to the degree of degradation of the park and frequent livestock incursions, cattle also were a main prey item, comprising 22% of the lion's diet (Tumenta et al., 2013). In Pendjari NP large wild prey comprised 36.7% of prey and buffalo (*Syncerus caffer*) was the most frequent prey followed by kob. Both species were taken proportionally to availability while hartebeest (*Alcelaphus buselaphus*) and waterbuck (*Kobus ellipsiprymnus*) were preferred, i.e. their proportion in the diet exceeded their proportion in the available prey (Sogbohossou & Funston, 2011).

In southern Africa, lions prefer large, non‐mega‐herbivore prey (92–632 kg) (Clements et al., 2014; Hayward & Kerley, 2005), while the weight range for most taken prey is lower than for preferred prey and ranges between 40 and 251 kg (Hayward & Kerley, 2005). Springbok (*Antidorcas marsupialis*) and Buffon's kob resulted the most killed species in the areas where they were present, but taken less than expected compared to availability, i.e. avoided. Blue wildebeest (*Connochaetes taurinus*), gemsbok (*Oryx gazella*) and buffalo followed as most taken prey, and these species were preferred as compared to availability (Hayward & Kerley, 2005).

Prey biomass, in migratory systems more specifically lean season biomass, has a direct influence on carnivore densities and can be used to calculate the ecological carrying capacity of an area for its carnivores (Bauer et al., 2008; Hayward et al., 2007; Loveridge & Canney, 2009; Van Orsdol et al., 1985). Studying local differences in prey preferences is needed to understand how predators adapt their diet to different environmental factors and changes in relative abundance of prey as this in turn will influence prey population as well as the social structure, home range and interspecific competition of carnivores (Bauer & de Iongh, 2005; Creel et al., 2018; Loveridge et al., 2009; Van Orsdol et al., 1985). Thus understanding of local prey preferences is important to guide management actions.

Zakouma NP (Chad) is the only savannah park in Central Africa where large herbivore biomass has not declined (Scholte et al., 2022), but rather showed an exponential increase for many species, especially in the last decade (Fraticelli et al., 2021). Zakouma NP was created in 1963, with governmental management until 2010 when the Government of Chad signed a public‐private partnership agreement with African Parks for the management of Zakouma NP, which was extended to the surrounding Greater Zakouma Ecosystem in 2017. Under African Parks management the budget increased, doubling between 2011 and 2018 and almost doubling again with the addition of the Greater Zakouma Ecosystem, exceeding 6 M$ annually (African Parks, 2011, 2018, 2019). Law enforcement was improved from previous practice by increasing the number of rangers, organizing regular training, establishing a Rapid Response Unit, and maintaining field presence year‐round, as well as improving communication between teams with VHF technology and with communities with an extensive intelligence network (African Parks, 2013). This led to improved security, decreased poaching and increased abundance of prey (African Parks, 2021).

A study on lion prey preference has been previously carried out in Zakouma NP between 2003 and 2006 (Vanherle, 2011). The results reported elephant (*Loxodonta africana*) calves as the most frequent carcasses, followed by buffalo, waterbuck and hartebeest, but only waterbuck was significantly preferred (Bauer et al., 2008; Vanherle, 2011).

**FIGURE 1:**
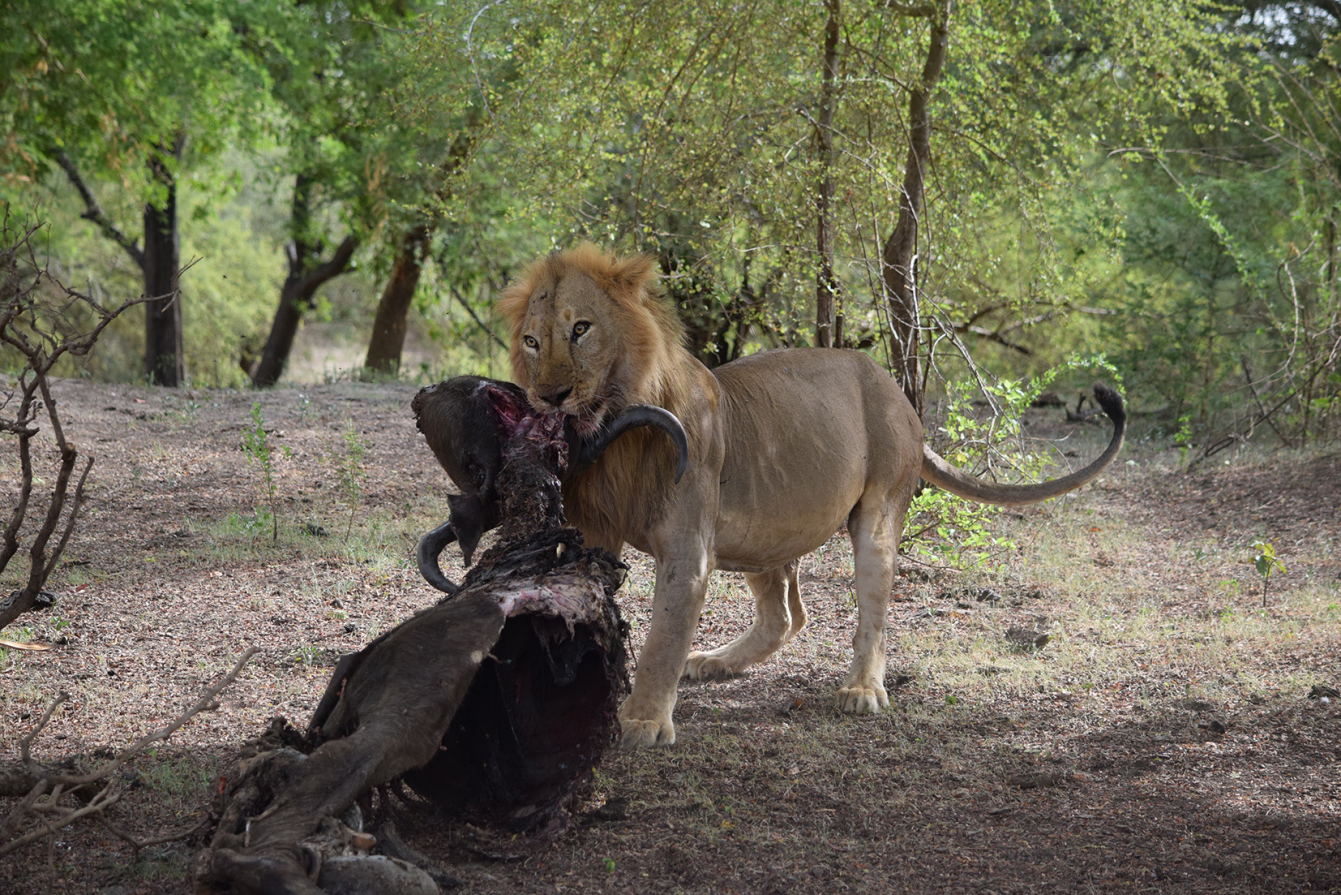
Male lion on a buffalo kill in Zakouma National Park, July 2022. Photo credit: Abdoulaye Abakar Zayed.

In this study we explore how changes in management in Zakouma NP, have influenced prey selection by lions. We collected lion feeding observations in Zakouma NP between January 2020 and June 2023 through opportunistic observations of lions on kills (Figure 1) and GPS clusters from collared lions. We expected a shift away from elephant predation, and an increase in predation and preference of other large herbivores, similar to other savannah ecosystems not suffering from prey depletion.

Finally, we also used the information on prey biomass to estimate carrying capacity with lion‐prey biomass equations from the literature and compare the performance of these estimates for this park in Central Africa with effective management.

## 2 MATERIALS AND METHODS

### 2.1 Study area

Zakouma National Park is a 3050 km^2^ protected area in southern Chad (19.350,10.568: 19.999,11.077), situated in a larger pastoral landscape of >28,000 km^2^ that incorporates also the Bahr Salamat and Siniaka Minia faunal reserves and the wildlife corridors around Zakouma NP (African Parks, 2021). The climate is Sudano‐Sahelian, characterized by three seasons: wet season (June–October), with an average rainfall of 800 mm; cold dry season (November to February), characterized by relatively low night temperatures and still widespread surface water; and hot dry season (March to May), with a limited number of waterholes available.

Zakouma National Park is also characterized by the wet season migration of some wildlife species, including elephants (Dolmia et al., 2007; Labuschagne, 2014), hartebeest and tiang (*Damaliscus lunatus*) (Poilecot, 2006). Other species, such as giraffe, roan and lion, show seasonal movements but over shorter distances, either moving to other areas inside the park or in the area surrounding the park (Clark et al., 2023; Fraticelli et al., submitted).

Zakouma's large herbivore numbers, have increased since the mid‐1980s (Scholte et al., 2022) and now the park sustains robust and still growing prey populations (Fraticelli et al., 2021). Only elephants are still well below historic density and recovering from the heavy poaching of the early 2000s, due to their long generation time and slow reproduction.

A study on lion ecology was conducted by Vanherle (2011) between 2003 and 2006 during a period of heavy poaching and when wildlife numbers were still low, with the same methods as the current study, namely by recording prey species when lions were spotted while feeding during monitoring drives. It showed an avoidance of buffalo and tiang, a preference for waterbuck and a mean group size of 2.7 lions (Bauer et al., 2008; Vanherle, 2011).

### 2.2 Data collection

Between February 2020 and June 2023, routine lion monitoring drives were carried out in Zakouma National Park in the dry season (November to June). On average, five monitoring drives were carried out each week, with fewer drives in the early (November, December) and late season (June) when ungraded roads and rains prevented movement. Morning drives started around 6 a.m. and lasted 4.0 ± 1.6 h, while afternoon drives started around 15:30 and lasted 3.0 ± 1.4 h. All instances of lions feeding on a carcass were recorded with date and GPS point, as well as recording the species of the prey and the composition of the lion group. Additional sightings were collected by trained tourist guides and law enforcement teams and shared with the research team. We excluded the few carcasses that were known to be scavenged and not hunted by lions, this was the case when the carcass was observed prior to when lions were recorded, with no evidence of bite marks. This occurred especially in the late dry season when weakened animals found themselves stuck in the drying waterholes and died before predators detected their presence. Dates and coordinates of all records were compared to ensure no double counting occurred.

Five lions were collared in February 2020, and further collaring operations took place every dry season during the study period, for a total of 15 individual lions being tracked. Collaring operations are routine work under the contract of delegated management between African Parks and the Government of Chad (collaring permits: N°100/MEEP/DG/DGTRFFP/DCFAP/2019, N°107/PCMT/PMT/MEPDD/SG/DGRFFP/DFAP/2022). African Parks’ Standard Operating Procedures for Animal Handling and Care (African Parks, 2020) were followed during all operations (see Appendix 1 for more information).

On average 6 collars were active each field season (December to May), ranging from a minimum of 1 to a maximum of 12 collars active simultaneously each month of the study. GPS location clusters from collared lions were opportunistically checked to determine the presence and species of kills.

Availability of prey was derived from the 2021 dry season total aerial count of Zakouma National Park, which counts all mammals visible from the plane (>12 kg) and provides population estimates for most medium (>40 kg) and large (>250 kg) species (Fraticelli et al., 2021).

### 2.3 Analysis

We used Jacob's index to assess prey preference: $D=\frac{r-p}{r+p-2\mathrm{rp}}$, where *r* is the proportion of kills of a certain prey species over all kills and *p* is the number of individuals of that species over all available prey (Jacobs, 1974). This index has a minimum value of −1 for avoided prey and a maximum of 1 for preferred prey; we consider *D* < −0.3 as a threshold for species avoided and *D* > 0.3 as a threshold for species preferred, while species with an index −0.3 < *D* < 0.3 are killed in proportion to their availability (Laurenzi et al., 2016; Lazzaro, 1987; Lückstädt & Reiti, 2002; Rasphone et al., 2022).

We used different equations of lion‐prey biomass relationship from the literature (Bauer et al., 2008; Hayward et al., 2007; Loveridge & Canney, 2009; Van Orsdol et al., 1985) to estimate lion carrying capacity in Zakouma NP. Average female biomass was derived from Kingdon (2015), except for buffalos for which we used 473 kg as reported by Sogbohossou and Funston (2011) for Benin. African buffalos exhibit large variability in morphology across the continent and Kingdon (2015) reports a large weight range for buffalos (425–850 kg). We could not find exact measures for buffalos of the same subspecies as Zakouma's (*S. c. aequinoctialis*), but anecdotal reports suggest they are rather smaller than Cape buffalo and more in the range of buffalos in Benin (*S. c. brachyceros*) so we used their biomass to more closely approximate the weight of Zakouma's subspecies (Smitz et al., 2013). Biomass for food intake was calculated as 75% of average female biomass.

## 3 RESULTS

A total of 156 lion kills were recorded; 134 from direct observation and 22 from lion collar GPS cluster checks. We excluded 8 records where the prey was not identified, 2 records of livestock, and 5 records of small prey species including: African civet (*Civettictis civetta*), patas monkey (*Erythrocebus patas*), olive baboon (*Papio anubis*) and unidentified bird. The mean lion group size as observed on the kills in this study was 3.6 ± 2.7 lions (2.5 ± 1.7 adults).

Large (>250 kg) prey species constituted 61% of kills. Buffalos constituted 46% of kills providing 57% of total kill biomass. Giraffe (*Giraffa camelopardalis*) provided another 29% of biomass so that these two larger prey species constituted 86% of lion diet. Waterbuck followed with 7% and hartebeest with 3% and warthog (*Phacochoerus africanus*) with 2%, while all other species contributed 1% or less (Table 1).

**TABLE 1:** Number of kills and corresponding Jacob's index for prey species, separated into all kills year round, cold dry season (December to February) and hot dry season (March to May).

Species		Average adult female weight	All kills		Cold dry season		Hot dry season	
Common name	Scientific name		Number of kills	Jacob's index	Number of kills	Jacob's index	Number of kills	Jacob's index
Giraffe	*Giraffa camelopardalis*	876	18	0.45	6	0.45	8	0.34
African buffalo	*Syncerus caffer*	473	65	−0.14	22	−0.12	38	−0.10
Roan	*Hippotragus equinus*	251.5	3	−0.16	1	−0.16	2	−0.07
Waterbuck	*Kobus ellipsiprymnus*	180	21	0.28	4	−0.03	16	0.44
Hartebeest	*Alcelaphus buselaphus*	150.5	12	−0.21	5	−0.09	5	−0.36
Tiang	*Damaliscus lunatus*	112.5	3	−0.67	2	−0.42	1	−0.79
Kob	*Kobus kob*	68.5	2	−0.48	1	−0.30	1	−0.52
Warthog^a^	*Phacochoerus africanus*	60	14		5		6	
Reedbuck	*Redunca redunca*	40	2	−0.46	1	−0.29	1	−0.51
Common duiker^a^	*Sylvicapra grimmia*	16	1		0		1	

^**a**^ No population estimates available for these species.

Our wet season prey availability and lion kill data were insufficient to calculate wet season preferences. Based on collar data of migratory antelope species (African Parks, unpubl. data) we assume that most prey is resident inside the park from November through May, so population estimates from the aerial survey carried out at the end of April/early May (Fraticelli et al., 2021) reflect prey availability for both the cold dry and hot dry season.

The estimated ecological carrying capacity from three different equations gave similar results with 421, 379 and 382 lions in Zakouma, only the Loveridge and Canney (2009) equation for conflict areas deviated with an estimate of 121 lions (Table 2).

**TABLE 2:** Calculations of carrying capacity with different equations from the literature.

Equation	Reference	Biomass	Predicted lion density (/100 km^2^)	Predicted Zakouma lion abundance
*y* = 0.0109x ^0.8783 *r* ^2^ = 0.9271 *n* = 83	Loveridge and Canney (2009) equation for non‐conflict area	All prey biomass as average female weight	13.79	421
*y* = 3.39 + 0.0029x *r* ^2^ = 0.765 *p* < .0001 *n* = 23	Equation derived from data in Van Orsdol et al. (1985) and Bauer et al. (2008)	Lean season prey biomass, estimated as average female weight, excluding hartebeest and tiang	12.43	379
*y* = −2.158 + 0.377x *r* ^2^ = 0.626 *p* < .001 *n* = 23	Hayward preferred prey equation (Hayward et al., 2007)	Preferred prey (wildebeest, buffalo, gemsbok, giraffe, zebra), 75% adult female weight (Hayward & Kerley, 2005)	12.52	382
*y* = 0.0017x ^0.9532 *r* ^2^ = 0.7791 *n* = 29	Loveridge and Canney (2009) equation for conflict area	All prey biomass as average female weight	3.96	121

## 4 DISCUSSION

Methods to look at prey selection tend to be biased towards larger prey, where lions spend more time feeding and are thus more likely to be located during monitoring drives or through GPS clusters. Smaller prey is often eaten more quickly, decreasing the probability of detection (Bacon et al., 2011; Tambling et al., 2012). Smaller species are also more difficult to count, and their availability is especially underestimated in aerial counts (Jachmann, 2002). Due to its small size, warthog is plausibly impacted by detection bias and thus is likely to have a more relevant role in lion diet than reported here, where this species comprised 10% of the recorded prey kills, is the second most killed species among the medium‐sized prey, and fifth in terms of biomass contribution to lion diet. While the contribution of small prey may have been underestimated (Groen et al., 2023), the availability of larger prey in Zakouma seems determinant in the diet composition of lions (Barnardo et al., 2020; Tambling et al., 2012).

Due to the lower wildlife density (African Parks, unpublished data) and lower visibility in the west (higher grass, denser bush, fewer roads) most records were collected in the eastern part of Zakouma NP where wildlife concentrates in the dry season. The data collected is insufficient to test for spatial variation, but we have no reason to suspect that this influenced to any major extent the here presented results.

In this study we observed lions preying on livestock only on two occasions, both outside of the park when following a collared lion. Livestock depredation might be more important in the wet season, like in Waza NP (Bauer & de Iongh, 2005; Tumenta et al., 2013), but our data are insufficient to discuss the importance of human wildlife conflict.

Our results differ slightly from the results of the prey preference analysis (Bauer et al., 2008) ran on the data collected in 2003–2006 in Zakouma NP (Vanherle, 2011). In both the current study and the 2003–2006 data (Vanherle, 2011) buffalos were the most frequently occurring prey, but in the previous prey selection analysis (Bauer et al., 2008) they resulted as avoided, while in the present study they are killed proportional to availability. Kob were killed proportional to availability in the earlier study (Bauer et al., 2008) while they now come out as avoided, but in both cases kill numbers were low. Kob avoidance is in stark contrast to other protected areas of West and Central Africa where kobs form a large proportion of lion kills and are usually eaten proportionally to availability (Bodendorfer et al., 2006; Breuer, 2005; Ruggiero, 1991; Sogbohossou & Funston, 2011). Waterbuck used to be significantly preferred according to the analysis on the 2003–2006 data (Bauer et al., 2008) while current results show that this species is only preferred in the hot dry season and preyed upon proportionally to availability during the year. Tiang remains avoided in Zakouma, while in other parts of Africa topi/tsessebe, the closest relatives of tiang, are generally taken in accordance to availability (Hayward & Kerley, 2005).

Bauer et al. (2008) proposed that the abundance of larger prey in many degraded areas is too low to sustain large lion foraging groups, leading to a preference for the medium‐sized prey. Here we observed a reversal of this process following prey recovery in Zakouma NP, with a preference for larger prey and increased lion group size. Indeed the mean lion group size observed on the kills in this study was higher than reported for Zakouma in the previous study (Bauer et al., 2008) and also higher than the average group size in West and Central Africa (Bauer et al., 2008).

Only two records of lions feeding on giraffe were recorded in the older study (Vanherle, 2011), with one being a sick individual where the lions only ate the entrails, and the other being a calf. In our study, giraffe was a preferred prey species accounting for 13% of kills. Between 2005 and 2021 giraffe numbers have increased from an estimate 350 to an estimated 1546 (Fraticelli et al., 2021; Vanherle, 2011), this increase in abundance likely augmented the encounter rate between lions and giraffes, offering increased opportunities for lions to hunt this species and further indication of a return to larger prey when their population is restored.

Although not considered lion prey species, the older study recorded 12 instances of lions feeding on juvenile and sub‐adult elephants (Vanherle, 2011), while none were recorded in the present study, except a single instance of a lion briefly scavenging on a fresh elephant carcass. This uncommon behavior has been observed in other parts of Africa and attributed to low number of ungulate species during migration seasons (Power & Compion, 2009), vulnerability of dependent calves during low rainfall years, when herds need to move longer distances in search of food (Loveridge et al., 2006) or as a consequence of poaching, where calves are orphaned or abandoned and lack the protection of the herd, or animals already wounded by poachers (Ruggiero, 1991). In the early 2000s Zakouma underwent a period of heavy elephant poaching with numbers decreasing by almost 90%, from the 4351 estimated in 2002 (Poilecot, 2008) to the 454 counted in 2011 (Potgieter et al., 2011). After a substantial change in management in 2010, which included reinforced protection and improved community support, elephant poaching has steadily decreased and the last case of poaching of elephant in Zakouma was recorded in 2016 (African Parks, 2021). In retrospect, lion predation on elephants in the early 2000s in Zakouma seems to have been a temporary phenomenon of opportunistic prey choice.

When comparing the cold dry season and the hot dry season, hartebeest and waterbuck are the only species showing a shift: while in the cold dry season both are eaten in relation to their availability, in the hot dry season hartebeest are avoided while the waterbuck become preferred. Waterbuck are more water‐dependent than hartebeest, and thus likely more easily predated upon during the months of low surface water availability. A similar seasonal shift in secondary prey of lions was also recorded in Hwange NP (Zimbabwe) with a higher relative contribution of water‐dependent suidae and zebra (*Equus quagga*) to lion diet in the late dry season and a higher contribution of less water‐dependent species, such as kudu (*Tragelaphus strepsiceros*), giraffe and small‐antelopes in the early dry season when surface water is still widely available (Davidson et al., 2013).

Lions in West and Central Africa kill on average less large prey compared to southern and East Africa (Bauer et al., 2008), explained by the lower diversity and abundance of large prey species in this area (Craigie et al., 2010; Scholte et al., 2022). In Zakouma, which now has a high abundance of large prey, especially buffalo, lions tend to kill larger prey, 61% of kills being of large prey (>250 kg), adding up to 87% of the food biomass. Most authors use 250 kg as the cut‐off and include waterbuck as the medium‐sized prey, but if we follow Bauer et al. (2008) who put waterbuck in the category of large prey, we have a total of 76% of large lion kills in Zakouma compared to the 51% reported for West and Central Africa and the 65% for East and Southern Africa (Bauer et al., 2008).

The relevance of our work extends beyond West and Central Africa. Depletion of preferred large prey in Kafue NP (Zambia) caused a shift towards medium‐sized prey in the lion's diet, shifting from buffalo and hartebeest towards lechwe (*Kobus leche*) and warthog. Additionally it reduced diet breadth and increased prey niche overlap between carnivores (Creel et al., 2018). This shift was accompanied by changes in pride size: while maintaining survival rates comparable to other similar ecosystems, this population showed smaller prides and low recruitment (Vinks et al., 2021). We speculate that restoration of prey numbers in this ecosystem might also reverse lion prey preference and group size.

It is well known that significant relationships exist between prey biomass and carnivore density, and as such prey biomass can be used to calculate carrying capacity for large carnivores in a given area (Hayward et al., 2007; Van Orsdol et al., 1985). The most well‐known is the Hayward equation (Hayward et al., 2007), but this is not appropriate to estimate lion carrying capacity in most West and Central African sites due to the natural absence of three out of five species taken into consideration by the equation and the low abundance of the other two species. However, in our case it performed as well as the others, as the species contributing most to the diet of lions in Zakouma NP are buffalos and giraffe, as in Hayward and Kerley (2005) and Hayward et al. (2007).

Total prey availability corresponds to a carrying capacity of approximately 400 lions, but 70% less in a system where livestock conflict is prominent with an estimate of 121 lions (Table 2). While Zakouma NP is well protected, it is not fenced and is surrounded by community land with both permanent villages and transhumance corridors within 10 km of the park borders. Lions, like most other wildlife, move outside of the park in this area either regularly or seasonally, and they suffer from conflict with humans, ranging from bushmeat poaching bycatch to retaliatory killing (Fraticelli et al., submitted).

In conclusion, lion's prey selection in Zakouma NP is consistent with that of other intact protected areas, with larger foraging groups hunting larger prey. The difference with the results of the study carried out in 2003–2006 indicates that this is likely due to the increase in large prey species in the park as a result of more effective management after delegation to African Parks. Nonetheless, lion density is unlikely to reach the estimated carrying capacity due to the lack of a hard boundary between the national park and the surrounding community land, leading to multiple conflicts.

## AUTHOR CONTRIBUTIONS

**Chiara Fraticelli:** Conceptualization (lead); data curation (lead); formal analysis (lead); investigation (equal); methodology (lead); project administration (lead); resources (lead); writing – original draft (lead); writing – review and editing (equal). **Abdoulaye Abakar Zayed:** Investigation (equal); writing – review and editing (equal). **Herwig Leirs:** Conceptualization (supporting); supervision (equal); writing – review and editing (equal). **Hans Bauer:** Conceptualization (supporting); supervision (lead); writing – original draft (supporting); writing – review and editing (equal).

## CONFLICT OF INTEREST STATEMENT

The authors declare no conflict of interest.

## Data Availability

Count of kills used for the analyses in this study are available in Table 1. Any further information can be made available upon request from the corresponding author.

## APPENDIX 1 LION CAPTURE AND COLLARING PROCEDURES

### 1.1

All operations followed African Parks’ Standard Operating Procedures for Animal Handling and Care (African Parks, 2020). Government of Chad collaring permits: N°100/MEEP/DG/DGTRFFP/DCFAP/2019, N°107/PCMT/PMT/MEPDD/SG/DGRFFP/DFAP/2022.

Lions were approached by vehicle to a distance of 10–15 m during the early hours of the day or late afternoon, when ambient temperature is lower, to reduce the risk of overheating during immobilization. The target lion was darted with a Dan‐Inject gas powered dart gun using a combination of Zoletil (100 mg for males and 80 mg for females) and Medetomidine (6/8 mg for males and 4/5 mg for females). After 10–15 min from darting during which the position and breathing of the animal were monitored from a distance, and after checking that the lion was effectively immobilized, the animal was approached on foot and a blindfold was set to cover the eyes. After a quick assessment by the veterinarian to ensure the immobilization was safe, and approval to proceed, the team set the collar (African Wildlife Tracking, Pretoria, South Africa; Followit Tellus, Lindesberg, Sweden), took measurements of body size, pictures of teeth, whisker spots and other identifying features, and blood and/or hair samples. The reversal Atipamezole (×2 to ×3.75 Medetomidine) was given on average 1 h after darting, after which the team retreated in the car at a safe distance from which they could monitor the lion and at the same time not disturb it or make it feel threatened. The team remained with the lion until it was aware enough to be able to move a short distance away. In case the operation occurred late in the afternoon, the team remained longer to ensure the safety of the lion from other predators that might be active in the area. Special attention was given to the collar data of the lion in the days following such operations to confirm a return to normal activity.

Slight variations occurred in the procedure and drug doses depending on the veterinarian leading the operation and based on their experience and assessment of the situation.
